# Assessing the impact of road traffic on cycling for leisure and cycling to work

**DOI:** 10.1186/1479-5868-8-61

**Published:** 2011-06-10

**Authors:** Charlie E Foster, Jenna R Panter, Nicholas J Wareham

**Affiliations:** 1 Department of Public Health, University of Oxford, UK; 2 Medical Research Council Epidemiology Unit and Centre for Diet and Activity Research (CEDAR), Institute of Public Health, Cambridge, UK

**Keywords:** Cycling, traffic, GIS

## Abstract

**Background:**

To explore the relationship between leisure and commuter cycling with objectively measured levels of road traffic and whether any relationship was affected by traffic levels directly outside of home or in local neighbourhood.

**Findings:**

We conducted a secondary analysis of data from the UK European Prospective Investigation of Cancer (EPIC) Norfolk cohort in 2009. We used a geographical information system (GIS) and gender specific multivariate models to relate 13 927 participants' reported levels of cycling with an index of road traffic volume (Road Traffic Volume Index Score - RTVIS). RTVIS were calculated around each participants home, using four distance based buffers, (0.5 km, 1 km, 2 km and 3.2 km). Models were adjusted for age, social status, education, car access and deprivation. Both genders had similar decreases in leisure cycling as traffic volumes increased at greater distances from home (OR 0.42, (95% CI 0.32-0.52, p < 0.001) for women and OR 0.41, (95% CI 0.33-0.50, p < 0.001) for men in the highest quartile at 3.2 km). There was no effect of traffic volumes at any distance on commuter cycling.

**Conclusions:**

Traffic volumes appear to have greater impact on leisure cycling than commuter cycling. Future research should investigate the importance of traffic on different types of cycling and include psychosocial correlates.

## Background

Cycling is considered to be a healthy, low carbon and sustainable physical activity behaviour [1]. Although travel by bicycle does introduce health risks through accidents and injuries [2] the health benefits of cycling have been shown to outweigh these risks [3]. More specifically, studies have suggested that commuter cyclists have a lower mortality risk than non-cycling commuters, independent of physical activity levels [4]. Across many international countries the numbers of car trips are increasing and in some there are simultaneous declines in active travel trips by walking or cycling [5].

Characteristics of the built environment are also suspected to contribute to levels of walking and cycling [6], although for active travel behaviors in particular the evidence is inconsistent [7]. It could be hypothesized that in areas which have both unsupportive built environments for active travel and high traffic volumes, a double burden of negative associations for active travel could be produced. International studies have focused on the negative impact of perceptions of traffic levels and safety on walking and cycling but have not used objective measures of traffic volume [8,9].

In our previous study, exposure to higher levels of traffic around the home was associated with less leisure cycling [10]. The aim of this study was to (i) explore the relationship between leisure cycling and commuter cycling with objectively measured levels of road traffic derived using a geographical information system (GIS) and (ii) investigate if any relationship was affected by traffic levels directly outside of the home or between home and local destinations.

## Methods

The UK EPIC-Norfolk study was designed as a prospective cohort study and the methods of recruitment, sampling and overall sample representativeness have been described elsewhere [11]. Data on self-reported measures of physical activity was collected from 15,786 adults using EPAQ2 between 1998 and 2000. This questionnaire asks about the frequency and duration of physical activity at home, at work (including travel to work) and for recreation, over the past 12 months. Ethical approval for the EPIC-Norfolk study was given by the Norfolk Research Ethics Committee.

Participants were asked to report how often they used a bicycle to get to work using the response categories of 'always', 'usually', occasionally' and 'never or rarely'. Participants were classified as commuter cycling if they reported 'always' travelling to work by bicycle. Leisure cycling was assessed by three items which asked about the number of occasions of cycling for pleasure, for racing and rough terrain cycling. If participants reported at least 1 occasion of any of these activities they were classified as engaging in 'some' leisure cycling.

Objective measures of road traffic volume were estimated using a GIS (ESRI ArcGIS 9.2). We calculated a proxy measure of traffic volume (Road Traffic Volume Index Score - RTVIS) for each participant using four different distance based buffers around each individuals home postcode (0.5 km, 1 km, 2 km, 3.2 km). It was calculated by computing the total lengths of four different types of road (principal roads or motorways, A-roads (major roads), B-roads (minor or local roads) and unclassified roads) within these buffers (centred on participants' homes) and weighting these based on the average road speed for each classification [12]. Scores were divided into quartiles and the lowest quartile was used as the reference group. Using a variety of distance buffers allowed us to examine any potential differences in the associations between cycling behaviour and traffic volume at different proximities, as there is currently uncertainty about size of the area from home which influences commuting or leisure related activities. The choice of the largest radius size reflected the UK government's aim to encourage a shift from car use to walking or cycling for short journeys under 2 miles (3.2 km) [13]. We estimated that a 2 mile cycle journey (at 8 mph) should take an adult approximately 15 minutes.

Possible confounders included age, gender, social status, educational qualifications, area socioeconomic deprivation, car ownership, ethnicity, and self-reported health conditions.

We built a series of gender specific multivariate models to calculate the odds ratios of commuter and leisure cycling associated with RTVIS adjusted for identified confounders. We checked for effect modification and interactions between variables at each stage of the model [14].

## Results

Physical activity data were available for 15 572 participants, however we excluded those who had incomplete postcode data or had moved out of the study area (5.9%), unusually high levels of self reported physical activity (1.5%) and missing socio-demographic data (3.1%). This left 13 927 participants for analysis.

Table 1 shows the characteristics of the sample. A higher proportion of men reported any leisure cycling compared to women, however we found a slightly higher proportion of women reported any occasions of commuter cycling than men (p < 0.05).

**Table 1:** Characteristics of participants by socio-demographic, physical activity and environmental variables

	Men (n = 6134)				Women (n = 7793)			
	Occasions of leisure cycling		Occasions of commuter cycling		Occasions of leisure cycling		Occasions of commuter cycling	
	None	Any	None	Any	None	Any	None	Any
**Percentage of Subjects**	80.1	19.9	96.2	3.8	85.1	14.9	95.7	4.3
**Age**								
41-50 years of age	63.9	36.1*	91.4	8.6*	70.1	29.9*	91.5	8.5*
51- 60 years of age	73.0	27.0	93.4	6.6	80.4	19.6	93.2	6.8
61-70 years of age	82.7	17.3	97.6	2.4	893.	10.7	97.9	2.1
71-80 years of age	91.6	8.4	99.5	0.5	95.5	4.5	99.4	0.6
**Social status**								
Professional	75.4	24.6*	95.0	5.0*	82.8	17.2*	95.2	4.8*
Managerial & Tech.	80.0	20.0	97.7	2.3	84.0	16.0	97.0	3.0
Skilled non-manual	80.6	19.4	97.1	2.9	88.9	11.1	97.3	2.7
Skilled manual	80.1	19.9	95.1	4.9	83.9	16.1	94.9	5.1
Partly skilled	82.2	17.8	93.3	6.7	85.5	14.5	92.4	7.6
Unskilled	85.1	14.9	94.2	5.8	84.2	15.8	91.0	9.0
**Educational qualifications**								
Degree or higher	76.1	23.9*	95.9	4.1	80.4	19.6*	95.3	4.7
Any qualifications	78.9	21.1	96.4	3.6	83.3	16.7	96.3	3.7
No qualifications	85.0	15.0	95.7	4.3	88.2	11.8	95.5	4.5
**Car ownership**								
Yes	80.7	19.3*	97.3	2.7*	85.0	15.0	97.0	3.0*
No	75.7	24.3	88.2	11.8	85.2	14.8	91.0	9.0
**Townsend index**								
Quintile 1 (most affluent)	80.2	19.8	97.8	2.2*	86.2	13.8*	96.9	3.1*
Quintile 2	79.3	20.7	96.3	3.7	86.4	13.6	97.1	2.9
Quintile 3	78.6	21.4	96.4	3.6	82.2	17.8	95.7	4.3
Quintile 4	80.5	19.5	96.3	3.7	83.9	16.1	95.9	4.1
Quintile 5 (most deprived)	81.9	18.1	93.9	6.1	86.5	13.5	93.1	6.9
**Self-reported health**								
With condition	82.3	17.7*	97.4	2.6*	86.8	13.2*	96.1	3.9
Without condition	78.4	21.6	95.2	4.8	83.0	17.0	95.4	4.6

* Signifies significant trend across variable categories at *P *< .05.

Table 2 presents the adjusted odds ratios for reporting leisure and commuter cycling.

**Table 2:** Odds ratios (95% CI) for reporting leisure and commuter cycling in past month by quartiles of Road Traffic Volume Index Score

	Odds of leisure cycling (95% CI) a		Odds of commuter cycling (95% CI) b	
	Men	Women	Men	Women
**RTVIS within 500 m**				
Quartile 1 (Light Traffic)	1.00	1.00	1.00	1.00
Quartile 2	1.11 (0.92-1.34)	1.11 (0.92-1.34)	0.73 (0.50-1.07)	1.14 (0.82-1.57)
Quartile 3	1.80 (0.98-1.42)	1.16 (0.97-1.40)	0.71 (0.48-1.04)	1.06 (0.76-1.50)
Quartile 4 (Heavy Traffic)	1.23 (1.02-1.48)	1.05 (0.88-1.26)	0.76 (0.53-1.12)	1.20 (0.87-1.66)
**RTVIS within 1000 m**				
Quartile 1 (Light Traffic)	1.00	1.00*	1.00	1.00
Quartile 2	0.89 (0.74-1.06)	0.71 (0.60-0.85)	0.80 (0.50-1.28)	1.27 (0.89-1.81)
Quartile 3	0.73 (0.61-0.88)	0.47 (0.39-0.57)	1.60 (1.06-2.39)	1.37 (0.97-1.94)
Quartile 4 (Heavy Traffic)	0.49 (0.40-0.60)	0.48 (0.39-0.58)	1.27 (0.83-1.93)	1.01 (0.70-1.46)
**RTVIS within 2000 m**				
Quartile 1 (Light Traffic)	1.00*	1.00*	1.00	1.00
Quartile 2	0.83 (0.70-0.99)	0.68 (0.57-0.80)	1.08 (0.69-1.70)	1.44 (1.01-2.04)
Quartile 3	0.68 (0.56-0.81)	0.51 (0.42-0.61)	1.64 (1.07-2.50)	1.34 (0.94-1.91)
Quartile 4 (Heavy Traffic)	0.43 (0.35-0.53)	0.43 (0.35-0.52)	1.53 (1.00-2.34)	1.10 (0.76-1.60)
**RTVIS within 3200 m**				
Quartile 1 (Light Traffic)	1.00*	1.00*	1.00	1.00
Quartile 2	0.77 (0.65-0.92)	0.71 (0.60-0.84)	0.86 (0.54-1.36)	1.67 (0.17-2.36)
Quartile 3	0.61 (0.51-0.74)	0.47 (0.39-0.57)	1.58 (1.04-2.37)	1.14 (0.78-1.65)
Quartile 4 (Heavy Traffic)	0.41 (0.33-0.50)	0.42 (0.35-0.52)	1.31 (0.86-2.00)	1.11 (0.76-1.61)

*p value test for trend < 0.001

a Models adjusted for age, social status, education, car ownership, travel mode to work, occupational physical activity.

b Models adjusted for age, social status, car ownership, area deprivation, occupational physical activity and recreational physical activity

CI, Confidence Intervals; RTVIS Road Traffic Volume Index Score.

Using a 500 m buffer, an increasing RTVIS was associated with higher odds of leisure cycling and women's commuter cycling. Yet, with larger buffer sizes the odds of leisure cycling decreased with increasing RTVIS, however there were no such associations observed for commuter cycling for either genders.

## Conclusions

Exposure to increasing level of traffic around home was associated with a reduction in leisure cycling and not for commuter cycling. Both genders had similar decreases in leisure cycling as traffic volumes increased between 500 m to 1000 m from home.

A few studies have reported conflicting associations between characteristics of the built environment, traffic and different types of cycling behaviour [8,15,16]. However direct comparison is difficult due to differences in methods in construction of the outcome variable by combining walking and cycling, or all cycling or cycle path use. One case study of non-cyclists reported similar impacts of traffic made cycling dangerous based on a combination of the poor quality of the road environment, plus heavy and speeding traffic, and worries about the dangers of cycling [17].

Triano and Freedson recently identified that a diversity of non-comparable methods are a limitation to environmental/behavioral research [18]. This study was limited by use of a non-objective physical activity measure however data were collected before the possible application of such measures could be realistically used in such a large sample. Outcome variable data were collected using appropriate methods for a large cohort study, using a reliable and valid measure. The GIS derived exposure measure is based on road transport network and local road speed, which could easily be adopted by other researchers to make cross-study and country comparisons possible. The use of increasing buffer areas for traffic also avoided any potential inter-individual variation in the size of neighbourhood and warrants further research into what is the size of the local neighbourhood in conjunction with cycling [19]. Ideally it would be helpful also include measures of cycle path availability, alongside data on route choices by cyclists, who might choose to use roads with lower traffic volumes while cycling.

Future research should investigate the importance of traffic and include other possible psychological journey related correlates (e.g. attitude, confidence to cycling), by gender and age [20]. Models should consider the impact of environmental correlates at different distance around home for different types of cycling (commuting or leisure).

## Authors' contributions

CF & JP conceived of the study, and participated in its design and coordination and helped to draft the manuscript. NW participated in its design and coordination and helped to draft the manuscript. All authors read and approved the final manuscript.

## Conflict of Interest

The authors declare that they have no competing interests.
